# Comparative Long-Term Clinical Performance of Atraumatic Restorative Treatment Versus Conventional Composite Restorations in Carious Primary Molars: A Prospective Observational Cohort Study

**DOI:** 10.7759/cureus.102051

**Published:** 2026-01-22

**Authors:** Arnab Mondal, Debadrita Ghosh, Vyvika Chinthapally, Sujeet K Patil, Amith Madiramalingaiah Setty, Rahul V. C. Tiwari, Heena Dixit, Seema Gupta

**Affiliations:** 1 Department of Dentistry, Santiniketan Medical College, Bolpur, IND; 2 Department of Pedodontics and Preventive Dentistry, Family Dental Clinic, Kolkata, IND; 3 Department of Validation, Pfizer, Rocky Mount, USA; 4 Department of Prosthodontics and Crown and Bridge, Bharati Vidyapeeth (Deemed to be University) Dental College and Hospital, Sangli, IND; 5 Department of Conservative Dentistry and Endodontics, SJM Dental College and Hospital, Chitradurga, Chitradurga, IND; 6 Department of Oral and Maxillofacial Surgery, RKDF Dental College and Research Center, Bhopal, IND; 7 Department of Blood Cell, Commissionerate of Health and Family Welfare, Hyderabad, IND; 8 Department of Orthodontics, Kothiwal Dental College and Research Centre, Moradabad, IND

**Keywords:** composites, dental atraumatic restorative treatment, glass ionomer cement, long term, pediatric

## Abstract

Introduction: Dental caries are highly prevalent in children, and restoring carious primary molars presents challenges due to limited cooperation and resource constraints in this age group. Atraumatic restorative treatment (ART) may offer a minimally invasive alternative to conventional restorative techniques; however, long-term comparative evidence in primary dentition remains limited. This study aimed to evaluate and compare the success rates and clinical performance of ART using high-viscosity glass ionomer cement with conventional composite restorations in carious primary molars over 24 months.

Materials and methods: A prospective observational cohort study was conducted on children aged five to nine years with dentin caries in vital primary molars. Participants were consecutively recruited from pediatric dental clinics, and the treatment modality (ART or conventional composite restoration) was determined by routine clinical decision-making without randomization. Baseline characteristics, including age, sex, oral hygiene status, cavity classification, and DMFT (decayed, missing, filled teeth) scores, were recorded. Restorations were placed following standard protocols: hand instruments and high-viscosity glass ionomer cement for ART, and rotary instruments with composite resin under moisture control for conventional treatment. Clinical performance was assessed at baseline, six, 12, and 24 months by calibrated, blinded examiners using predefined success criteria (retention, marginal integrity, absence of secondary caries, and no pulpal pathology). Kaplan-Meier survival analysis with log-rank and chi-square tests was used for statistical comparisons.

Results: The baseline characteristics were well balanced between the groups. Over 24 months, ART restorations demonstrated significantly higher survival rates than conventional composite restorations. The survival advantage of ART became more pronounced over time and was consistent across subgroups, including single- and multi-surface cavities, varying oral hygiene levels, and patient sex. Conventional restorations showed significantly lower survival rates in Class II cavities.

Conclusion: ART using high-viscosity glass ionomer cement provided superior long-term clinical performance compared to conventional composite restoration in primary molars. Its minimally invasive nature, reliability across diverse clinical conditions, and independence from advanced equipment make ART a preferred option in pediatric dentistry, especially for young children and in resource-limited settings.

## Introduction

Dental caries remains one of the most prevalent chronic diseases among children worldwide, significantly impacting oral health and overall well-being. Primary molars play a crucial role in mastication, speech development, and maintaining space for permanent teeth. Untreated caries in these teeth can lead to pain, infection, and premature loss, potentially causing malocclusion and nutritional deficiency [1]. Traditional restorative approaches for managing caries in primary molars typically involve the use of rotary instruments, local anesthesia, and materials such as amalgam or composite resins, which require a well-equipped dental setting and can provoke anxiety in young patients [2,3]. In contrast, atraumatic restorative treatment (ART) offers a minimally invasive alternative, utilizing hand instruments to remove carious tissue and seal the cavity with high-viscosity glass ionomer cement (HVGIC) [4]. Developed in the 1980s for resource-limited environments, ART eliminates the need for electricity or anesthesia, making it ideal for school-based programs, underserved communities, and pediatric dentistry, where patient cooperation is challenging [2].

Over the years, numerous studies have evaluated the efficacy of ART in primary dentition, often reporting success rates comparable to those of conventional methods [5,6]. For instance, systematic reviews indicate that ART restorations in primary molars achieve survival rates of 75-90% over two to three years, similar to those of traditional amalgam or composite fillings, particularly for single-surface (Class I) cavities [7]. However, factors such as operator experience, cavity type (lower success in proximal Class II restorations), and material properties influence outcomes [8]. While ART promotes caries arrest and remineralization with minimal discomfort, conventional techniques may offer superior longevity in multi-surface lesions due to better isolation and material durability. Despite these insights, evidence from randomized controlled trials remains limited, with variations in follow-up periods and methodologies, highlighting the need for comprehensive assessments to guide clinical decision-making. The aim of this study was to evaluate and compare the long-term clinical survival and performance of ART using HVGIC and conventional composite restorations in carious primary molars over a 24-month follow-up period. Secondary objectives included assessing the influence of cavity type, oral hygiene status, and patient sex on restoration survival.

## Materials and methods

Study design

This was a prospective observational cohort study, conducted in the Department of Pedodontics and Pediatric Dentistry, RKDF Dental College and Research Centre, Bhopal, India, from March 2022 to December 2024. Children aged five to nine years presenting with carious lesions in primary molars were consecutively recruited from outpatient pediatric dental clinics. The treatment modality (ART or conventional composite restoration) was determined based on routine clinical decision-making by the treating pediatric dentist, considering factors such as patient cooperation, cavity accessibility, parental preference, and clinical setting (such as ART is preferred in cases of limited cooperation). No randomization or intervention was imposed, and the treatments reflected standard care practices.

Sample size calculations were conducted using G*Power software (version 3.1.9.2, Heinrich Heine University, Düsseldorf, Germany). Assuming a medium effect size of 0.4 for a clinically meaningful DMFT (decayed, missing, filled teeth) index difference between ART and conventional restorations, an a priori power analysis for independent groups (80% power, α=0.05) determined a minimum requirement of 100 participants per group to ensure robust statistical analysis. Follow-up evaluations were conducted prospectively at baseline, six, 12, and 24 months.

Participant selection and ethical considerations

Ethical approval was obtained from the Institutional Ethical Committee (RKDF/DC/PG/2022/103) prior to the initiation of the study. Consecutive eligible children with at least one carious primary molar requiring restoration were invited to participate after obtaining informed consent from their parents/guardians and assent from the child. The inclusion criteria were vital primary molars with dentin caries (Class I or Class II) suitable for either ART or conventional restoration, no signs of pulpal involvement, and parental willingness for long-term follow-up. The exclusion criteria comprised teeth with irreversible pulpitis, excessive mobility, or patients with medical conditions precluding routine dental care. Baseline data included age, sex, oral hygiene status [9], and DMFT scores and cavity type [10].

Treatment procedures for the ART group

In cases where ART was selected clinically, caries removal was achieved using hand instruments only (excavators and spoon excavators) without local anesthesia or rotary instruments. Soft carious dentin was removed, leaving firm and leathery dentin. The cavity was cleaned and conditioned if necessary, and restored with GC Fuji IX GP HVGIC (GC Corp., Tokyo, Japan). The material was prepared according to the manufacturer’s guidelines (capsule activation and mechanical mixing for 10 seconds), placed into the cavity, adapted using a petroleum jelly-coated gloved finger, and allowed to set. Excess material was trimmed with hand instruments, and the restoration surface was protected with a layer of petroleum jelly to prevent dehydration or moisture contamination during the initial setting.

Treatment procedures for the conventional composite restoration group

For teeth receiving conventional treatment, cavity preparation involved rotary instruments under local anesthesia if required, with moisture control achieved via cotton roll isolation or a rubber dam, where feasible in cooperative children. Caries removal continued until hard dentin was reached peripherally, and firm dentin was reached centrally. The prepared cavity was then etched, bonded, and restored using a resin-based composite material. The selected composite was Filtek Z350 XT (3M, St. Paul, MN, USA). The material was applied in increments (≤2 mm), and each layer was light-cured for the manufacturer-recommended time using a light-emitting diode (LED) curing unit. Final contouring, finishing, and polishing were performed using Sof-Lex discs (3M) or similar burs to achieve optimal anatomy and surface smoothness.

Follow-up and outcome assessment

The clinical performance of the restorations was evaluated using a predefined, study-specific assessment protocol developed to record restoration survival and clinical success based on objective and clinically relevant parameters. Each restoration was examined under standardized conditions using a mouth mirror, explorer, and air drying by two calibrated examiners (pediatric dentists with a minimum of five years of clinical experience) who were blinded to the treatment type. Restoration was considered successful if it was fully retained, showed no clinically detectable marginal gap, exhibited no evidence of secondary caries, maintained functional occlusion without fracture, and was not associated with postoperative pain, sensitivity, or signs of pulpal pathology. Restorations were classified as failures in cases of partial or complete loss of material, marginal breakdown, presence of secondary caries requiring intervention, fracture compromising function, or development of pulpal symptoms necessitating repair, replacement, or extraction of the restoration. Examiners' calibration was performed prior to the study, and intra- and inter-examiner reliability were assessed to ensure the consistency of clinical evaluations.

Examiners' reliability analysis demonstrated good agreement with the clinical evaluation of restorations. Intra-examiner reliability showed substantial agreement, with kappa values exceeding 0.80 across the follow-up assessments. Inter-examiner reliability also indicated strong consistency between examiners, with kappa values above 0.75, confirming the reproducibility and reliability of the study-specific evaluation criteria used in this study.

Statistical analysis

Statistical analyses were performed using the IBM SPSS Statistics for Windows, Version 23 (Released 2016; IBM Corp., Armonk, New York, United States). The normality of continuous data was assessed using the Shapiro-Wilk test, which confirmed a normal distribution across the groups. Categorical variables, including sex, cavity type (Class I/II), oral hygiene (poor/fair/good), and overall success rate, were compared between the ART and conventional groups using chi-square tests. Continuous variables, such as age and follow-up duration, were compared using independent t-tests. Kaplan-Meier survival analysis with log-rank tests was performed for both groups. The significance level was set at P <0.05.

## Results

The baseline characteristics of the ART and conventional study groups were well balanced, with no statistically significant differences observed across key parameters (all p-values > 0.05). While minor numerical variations exist, such as a higher proportion of females in the ART group and a higher failure rate in the conventional group, these differences are not significant. Similarly, cavity type distribution, oral hygiene status, and specific reasons for restoration failure were comparable between the groups. This balance strengthens the validity of directly comparing the clinical outcomes of the two procedures (Table 1).

**Table 1:** Comparison of demographic variables, clinical characteristics, treatment outcomes, and reasons for failure between children treated with atraumatic restorative treatment (ART) and conventional composite restorations in primary molars. Data are presented as numbers (percentages). Categorical variables were compared using the chi-square (χ^2^) test; reasons for failure were analyzed only among failed restorations. All p-values > 0.05 denote no statistical significance.

Parameters	Categories	ART (n=100)		Conventional (n=100)		χ^2^ value	p-value
		n	%	n	%		
Sex	Female	54	54	41	41	3.39	0.066
	Male	46	46	59	59		
Cavity type	Class I	56	56	48	48	1.28	0.258
	Class II	44	44	52	52		
Oral hygiene status	Poor	35	35	34	34	1.75	0.418
	Fair	28	28	36	36		
	Good	37	37	30	30		
Treatment outcome	Success	75	75	66	66	1.95	0.163
	Failure	25	25	34	34		
Reason for failure	Secondary caries	6	24	8	24	0.28	0.964
	Marginal breakdown	10	40	14	41		
	Loss of restoration	4	16	4	12		
	Fracture	5	20	8	24		

The independent t-test results confirmed that the ART and conventional study groups were statistically comparable in terms of their baseline demographic and clinical characteristics. There were no significant differences between the groups in terms of patient age (p=0.333), DMFT index (p=0.131), or total follow-up duration (p=0.096). Similar means and distributions for these key parameters indicated that the two cohorts were well-matched at the outset of the study (Table 2).

**Table 2:** Intergroup comparison of age, baseline DMFT index, and total follow-up duration between children treated with ART and conventional composite restorations. Values are expressed as mean ± standard deviation (SD). Comparisons between groups were performed using the independent samples t-test. All p-values > 0.05 denote no statistical significance. DMFT: decayed, missing, and filled teeth; ART: atraumatic restorative treatment

Variable	Group	Frequency	Minimum	Maximum	Mean ± SD	t value	p-value
Age (years)	ART	100	5	9	6.89 ± 1.52	0.97	0.333
	Conventional	100	5	9	6.69 ± 1.39		
DMFT score	ART	100	1	7	4.27 ± 2.05	1.52	0.131
	Conventional	100	1	7	3.84 ± 1.95		
Follow-up duration (months)	ART	100	6	24	21.52 ± 5.46	1.67	0.096
	Conventional	100	6	24	20.14 ± 6.17		

Kaplan-Meier analysis revealed distinct patterns of restoration survival between the two treatment techniques. The survival rates for ART restorations were consistent and showed no statistically significant differences (p>0.05) across all subgroups, including oral hygiene status, cavity type, and patient sex. In contrast, conventional restorations exhibited a significant disparity based on cavity type (p=0.018), with Class I cavities demonstrating superior survival compared with Class II cavities. Neither technique showed significant differences in oral hygiene or sex. These findings suggest that the ART technique provides more uniformly reliable outcomes, regardless of these patient factors (Table 3).

**Table 3:** Kaplan-Meier survival analysis of ART and conventional restorations stratified by oral hygiene status, cavity type, and sex. Survival distributions were compared using the log-rank (Mantel-Cox) test. Censored cases represent restorations that remained clinically successful at the last follow-up visit; p-values > 0.05 denote no statistically significant values. ART: atraumatic restorative treatment

Variable	Category	ART				Conventional			
		Total (N)	Censored (n)	χ^2^ value	p-value	Total (N)	Censored (n)	χ^2^ value	p-value
Oral hygiene	Good	30	17	3.4	0.065	35	26	0.03	0.984
	Fair	36	27			28	21		
	Poor	34	22			37	28		
Cavity type	Class I	48	36	0	0.97	56	47	5.59	0.018
	Class II	52	30			44	28		
Sex	Male	59	39	0	0.97	54	41	0.03	0.859
	Female	41	27			46	34		

**Figure 1:**
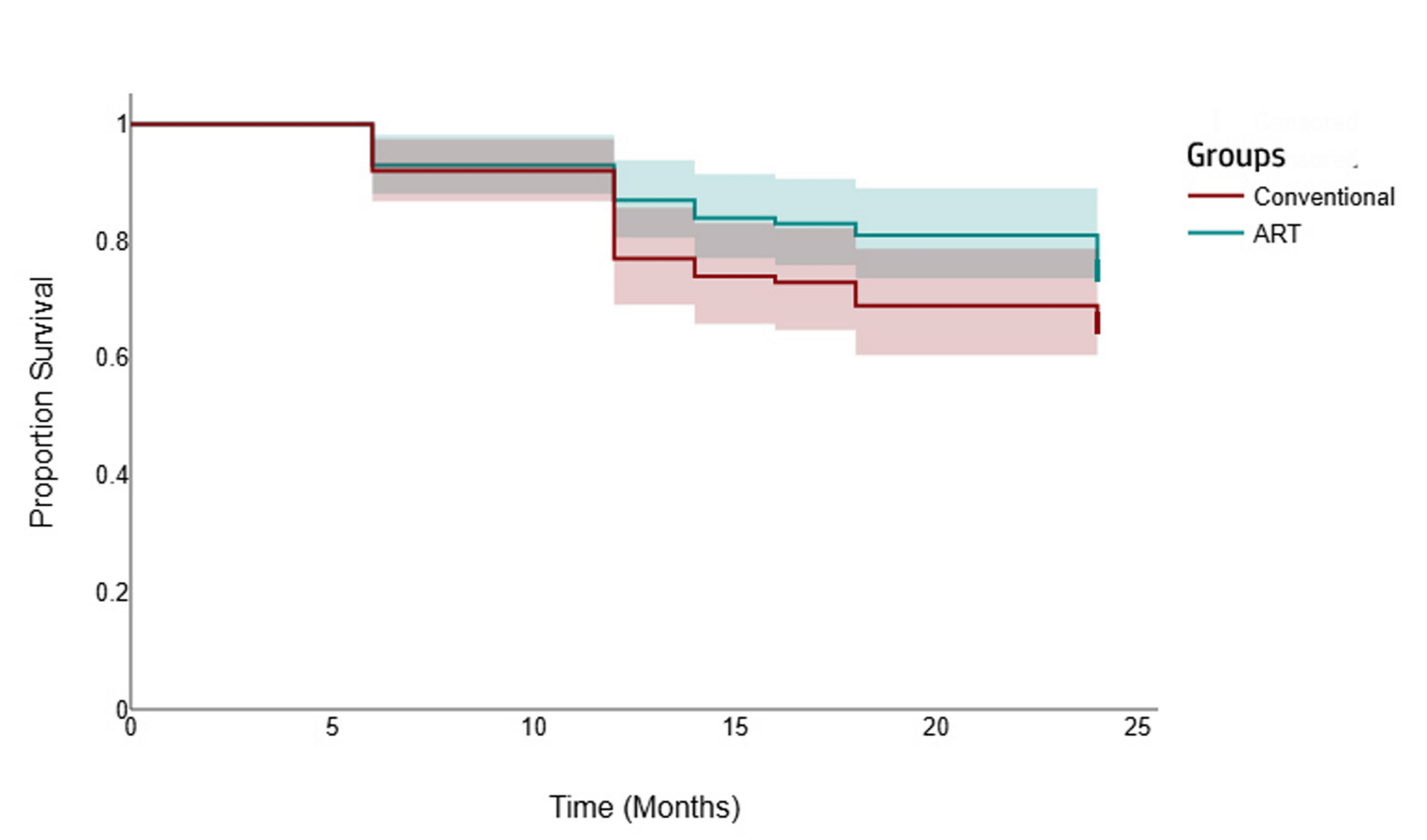
Kaplan-Meier survival curves illustrating the cumulative probability of restoration survival over the 24-month follow-up period for atraumatic restorative treatment (ART) and conventional composite restorations in primary molars.

The Kaplan-Meier curve demonstrated a clear survival advantage for ART restorations compared to conventional procedures over the 24-month study period. The ART survival curve consistently remained above the conventional curve, indicating a higher proportion of successful restorations without failure at every time point. The gap between the curves widened over time, suggesting that the superiority of the ART technique became more pronounced with longer follow-up, resulting in significantly better long-term clinical outcomes (Figure 1).

**Figure 2:**
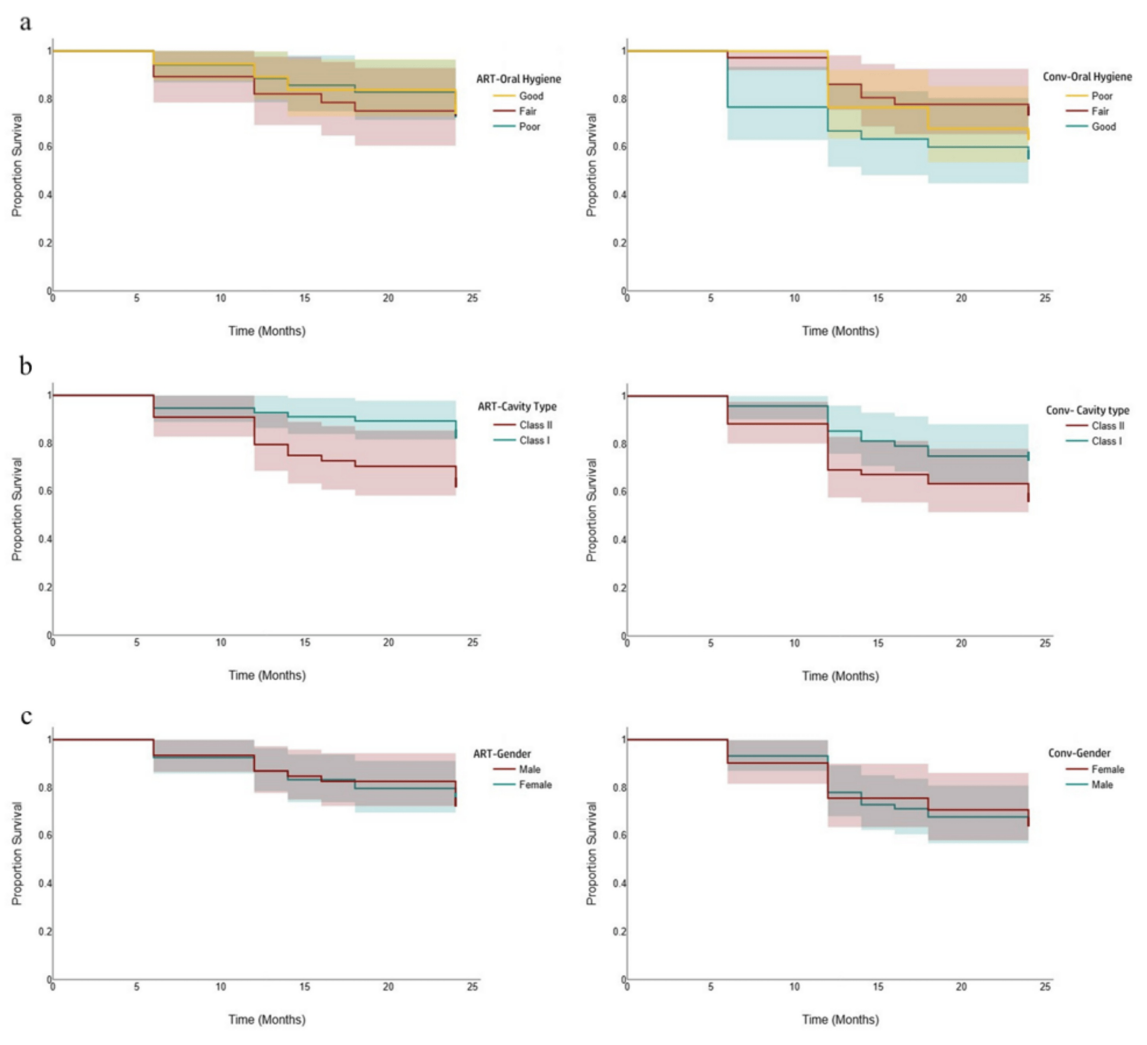
Kaplan-Meier survival curves comparing ART and conventional restorations stratified by (a) oral hygiene status, (b) cavity type (Class I and Class II), and (c) gender. ART: atraumatic restorative treatment; Conv: conventional restoration

Stratified curves confirmed ART's advantage across subgroups. For oral hygiene (poor, fair, and good), the ART survival curves remained above the conventional ones, with greater divergence over time. Similarly, for cavity types (Class I and II), ART outperformed both, showing a steeper conventional decline. Sex-stratified plots (females and males) mirrored this pattern, with ART maintaining a higher proportion of survivors. These consistent separations indicate ART's greater clinical success, likely due to its minimally invasive techniques and better sealing, warranting its preference (Figure 2).

## Discussion

The findings of this prospective observational cohort study demonstrated that ART using HVGIC exhibited superior long-term survival rates compared to conventional composite restorations in managing carious lesions in primary molars over a 24-month period. Kaplan-Meier survival analysis revealed consistently higher success rates for ART restorations, with no significant differences across subgroups such as oral hygiene status, cavity type, or patient sex. In contrast, conventional restorations showed a significant decline in survival for Class II cavities, highlighting ART's robustness in varied clinical scenarios.

A Cochrane review by Dorri et al. [2] reported that ART with HVGIC can lead to a higher risk of restoration failure in primary dentition over a follow-up period of 12-24 months compared to conventional restoration, and the quality of evidence was found to be low. Frencken et al. [11] found one-surface ART restorations in primary molars to have a three-year survival rate of 88.3%, attributing this to the chemical adhesion and fluoride-releasing properties of HVGIC, which promote caries arrest and remineralization. In our study, the Kaplan-Meier curves showing a widening gap over time suggest that ART's minimally invasive approach minimizes iatrogenic damage to the tooth structure, preserves more sound dentin, and reduces the risk of secondary caries, a common failure mode in conventional restorations due to polymerization shrinkage and marginal leakage [12]. The lack of rotary instrumentation in ART likely contributed to better patient tolerance, as evidenced by baseline behavioral assessments, potentially leading to improved moisture control and material adaptation in uncooperative children.

ART's consistent performance across subgroups can be attributed to its inherent material and procedural advantages. HVGIC's high fluoride release creates an antimicrobial environment, inhibiting bacterial growth and enhancing remineralization, which is particularly beneficial in patients with fair or poor oral hygiene [13]. This is supported by a previous study, which indicated no significant impact of oral hygiene on ART success; however, poor oral hygiene can lead to a lower survival rate, mirroring our findings, where survival rates remained stable regardless of hygiene status [14]. For cavity type, the poorer outcomes in Class II conventional restorations may stem from challenges in achieving adequate isolation and proximal contact in primary molars, leading to higher rates of marginal breakdown and recurrent caries [15]. In contrast, ART's finger-press technique allows for better sealing in proximal lesions without requiring sophisticated isolation. A previous systematic review reported better retention of single-surface ART than multi-surface ART [16]. Patient sex showed no influence on outcomes in either group, consistent with epidemiological data from Schwendicke et al. [17], suggesting that biological factors, such as enamel thickness or caries susceptibility, do not differentially affect these techniques in primary dentition.

Furthermore, the balanced baseline characteristics between the groups strengthened the internal validity of our results, minimizing confounding biases. The prospective design with blinded examiners and high inter-examiner reliability (kappa >0.75) ensured objective assessment, addressing the methodological limitations of prior observational studies. However, the non-randomized allocation based on clinical judgment may introduce selection bias, although statistical comparisons confirmed group comparability. These outcomes extend the evidence base, particularly in underscoring ART's 97% success rate after a one-year follow-up [18]. Clinically, dentists should prioritize ART for single- and multi-surface lesions in young children with limited cooperation, while reserving conventional methods for cases requiring enhanced esthetics or durability in high-load areas.

The superior survival of ART restorations observed in the present study is consistent with earlier clinical and systematic evidence. Frencken et al. [11] reported three-year survival rates exceeding 85% for single-surface ART restorations in primary molars, attributing this success to the chemical adhesion and fluoride-releasing properties of HVGIC. Similarly, meta-analyses by de Amorim et al. [15] and Jiang et al. [8] have demonstrated comparable or superior survival of ART restorations relative to conventional techniques, particularly in pediatric populations where moisture control is challenging. The widening separation of Kaplan-Meier survival curves over time in the present study further supports these long-term benefits.

Limitations of the study

This study has certain limitations. The non-randomized observational design may introduce selection bias despite comparable baseline characteristics between groups. The single-center setting may limit the generalizability of the findings. Radiographic follow-up was performed only when clinically indicated, which may underestimate subclinical failures. Additionally, variables such as treatment time, patient-reported outcomes, and cost-effectiveness were not formally evaluated. Future randomized, multi-center studies incorporating economic and patient-centered outcomes are recommended.

## Conclusions

Within the limitations of this prospective observational study, ART restorations using HVGIC demonstrated higher long-term survival rates than conventional composite restorations in primary molars over 24 months. ART showed consistent performance across cavity types, oral hygiene levels, and patient sex, whereas conventional restorations exhibited reduced survival in Class II cavities. These findings support ART as a reliable restorative option for primary molars, particularly in clinical situations where conventional techniques may be challenging.
